# Minority carrier decay length extraction from scanning photocurrent profiles in two-dimensional carrier transport structures

**DOI:** 10.1038/s41598-021-01446-5

**Published:** 2021-11-08

**Authors:** Yu-Chien Wei, Cheng-Hao Chu, Ming-Hua Mao

**Affiliations:** 1 Graduate Institute of Electronics Engineering, National Taiwan University, No. 1, Roosevelt Rd. Sec. 4, Taipei, 10617 Taiwan; 2 Department of Electrical Engineering, National Taiwan University, No. 1, Roosevelt Rd. Sec. 4, Taipei, 10617 Taiwan; 3 Graduate Institute of Photonics and Optoelectronics, National Taiwan University, No. 1, Roosevelt Rd. Sec. 4, Taipei, 10617 Taiwan

**Keywords:** Optical physics, Optical techniques

## Abstract

Carrier transport was studied both numerically and experimentally using scanning photocurrent microscopy (SPCM) in two-dimensional (2D) transport structures, where the structure size in the third dimension is much smaller than the diffusion length and electrodes cover the whole terminal on both sides. Originally, one would expect that with increasing width in 2D transport structures, scanning photocurrent profiles will gradually deviate from those of the ideal one-dimensional (1D) transport structure. However, the scanning photocurrent simulation results surprisingly showed almost identical profiles from structures with different widths. In order to clarify this phenomenon, we observed the spatial distribution of carriers. The simulation results indicate that the integrated carrier distribution in the 2D transport structures with finite width can be well described by a simple-exponential-decay function with the carrier decay length as the fitting parameter, just like in the 1D transport structures. For ohmic-contact 2D transport structures, the feasibility of the fitting formula from our previous 1D analytical model was confirmed. On the other hand, the application of a simple-exponential-decay function in scanning photocurrent profiles for the diffusion length extraction in Schottky-contact 2D transport structures was also justified. Furthermore, our simulation results demonstrate that the scanning photocurrent profiles in the ohmic- or Schottky-contact three-dimensional (3D) transport structures with electrodes covering the whole terminal on both sides will reduce to those described by the corresponding 1D fitting formulae. Finally, experimental SPCM on a p-type InGaAs air-bridge two-terminal thin-film device was carried out. The measured photocurrent profiles can be well fitted by the specific fitting formula derived from our previous 1D analytical model and the extracted electron mobility-lifetime product of this thin-film device is 6.6 × 10^–7^ cm^2^·V^−1^. This study allows us to extract the minority carrier decay length and to obtain the mobility-lifetime product which can be used to evaluate the performance of 2D carrier transport devices.

## Introduction

Successful development of nano-scaled semiconductor devices requires in-depth understanding of carrier transport behavior and other material properties^1,2^. Scanning photocurrent microscopy (SPCM) and the electron-beam-induced current (EBIC) have been widely used to extract carrier decay length or the depletion width in miniature structures such as nanowires with junctions^3,4^ and ohmic^5,6^ or Schottky^7^ contact. Carrier diffusion length extraction using SPCM or EBIC has been carried out in one-dimensional (1D) transport structures such as nanowires with a simple-exponential-decay function for p-n junctions or Schottky-contact devices. On the other hand, carrier decay length extraction for ohmic-contact nanowire devices requires a special 1D analytical model which has been established and confirmed by our previous work^6^. Furthermore, mobility-lifetime product also can be obtained from the minority carrier decay length.

**Figure 1:**
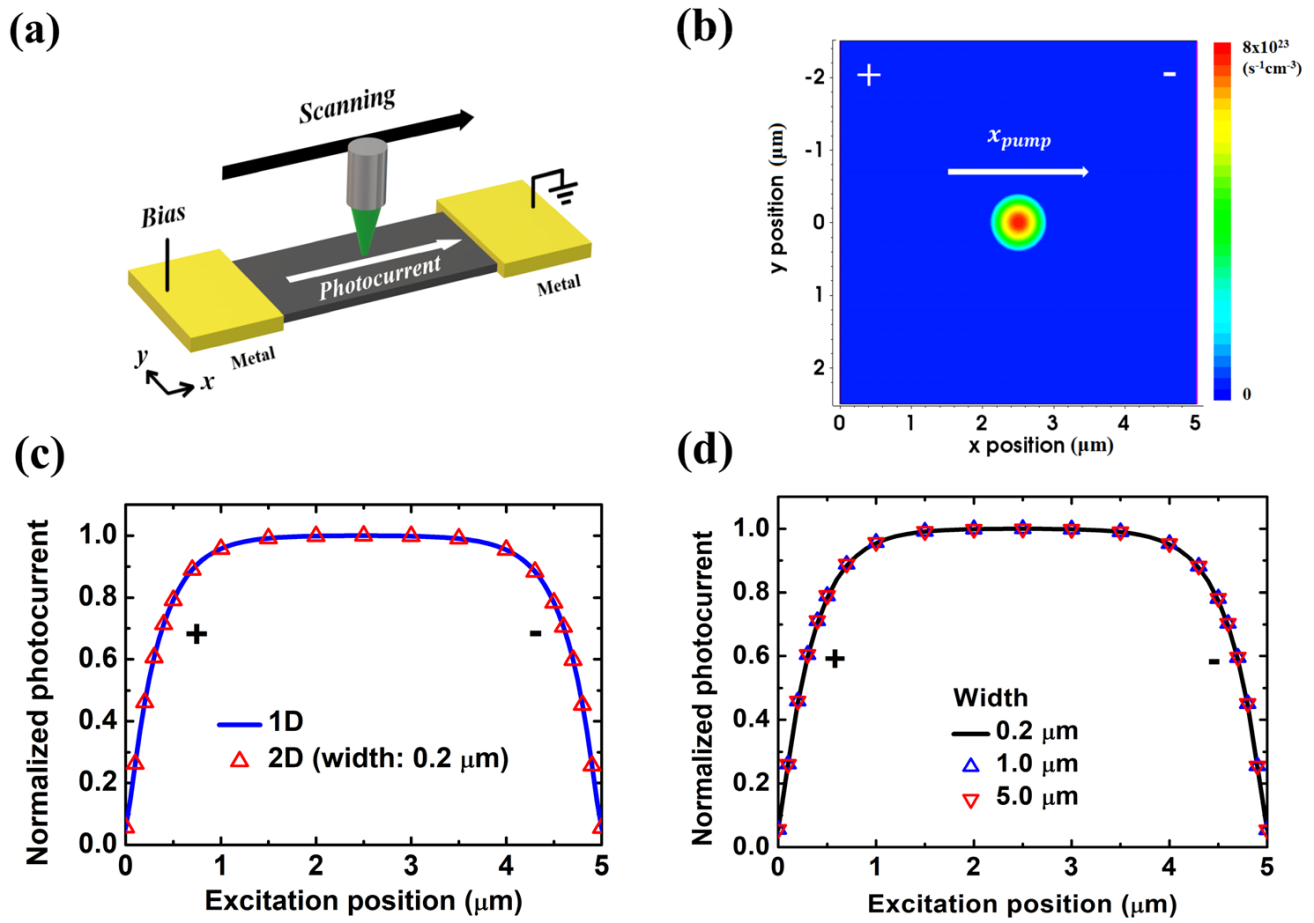
(**a**) Schematic of a SPCM setup in a two-terminal thin-film structure. (**b**) Mapping of 2D absorbed photon density per second, scanning across the channel. The anode is at $x = 0$ μm and the cathode is at $x = 5$ μm. (**c**, **d**) In ohmic-contact based devices. (**c**) Scanning photocurrent profile of the 0.2 μm wide case compared with that of the 1D transport structure. Our previous simulator was used in 1D simulation. (**d**) Scanning photocurrent profiles with different widths. *(**a**) is created using Microsoft Paint 3D ver. 6.2105.4017.0-URL: https://www.microsoft.com/en-us/p/paint-3d/9nblggh5fv99?cid=msft_web_chart.

SPCM and EBIC have also been applied in thin films^8–15^ and two-dimensional (2D) materials^16,17^ with p-n junctions or Schottky contact. Regarding ohmic-contact 2D transport structures, there have been no studies to extract carrier decay length either experimentally or numerically. As for 2D transport structures with p-n junctions or Schottky contact, there have been experimental reports in the literature^9,10,15^. In this paper, we studied SPCM numerically and experimentally in 2D transport structures, where the structure size in the third dimension is much smaller than the diffusion length and electrodes cover the whole terminal on both sides, as shown in Fig. 1a. To develop a fitting method for extracting minority carrier decay length in ohmic-contact transport devices and to justify the application of a simple-exponential-decay function for decay length extraction in Schottky-contact devices, numerical simulations in n-type InAs thin films were applied for the model verification. The experimental SPCM was applied on a p-type InGaAs air-bridge two-terminal thin-film device with ohmic contact. It was confirmed that the photocurrent profile can be well fitted by the specific fitting formula derived from our previous 1D analytical model^6^ and the electron mobility-lifetime product can be extracted.

## Results and discussion

In this work, a widely used and commercially available semiconductor device/process simulator Synopsys Sentaurus Technology Computer Aided Design (TCAD) was adopted for simulations. TCAD has been used specifically for studies on EBIC and field-effect transistors in the literature^12,18,19^. Here we used TCAD to simulate photocurrent under scanning laser excitation. A structural description of the thin-film structure is shown in Fig. 1b. The channel length $$L_{ch}$$ and the width of this two-terminal thin-film device are both 5 μm, where the anode is at $$x = 0$$ μm and the cathode is at $$ x = L_{ch}$$, both extending in the y direction to cover the whole terminal on both sides of the thin-film structure. The laser source pumps at the position $${\text{x}} = x_{pump}$$ and scans across the channel along $${\text{y}} = 0$$ μm. Its 2D Gaussian shape was utilized as the absorbed photon density profile, as shown in Fig. 1b, and the spot size is 100 nm. It should be noted that the spot size is defined as W of the Gaussian profile $${\text{A}}e^{{ - 2\frac{{\left( {x - x_{pump} } \right)^{2} }}{{W^{2} }}}}$$, where A is a constant^20^. In developing the analytic formulae for SPCM fitting, an ideal delta-function source was assumed^6^. However, in TCAD simulations, no delta-function source was allowed. Therefore, we chose the 100 nm spot size that is close to the assumption of an ideal delta-function source while it remains a finite-sized source in TCAD simulations. The absorbed photon density per second was calculated from the laser pumping power and 100% conversion of absorbed photons into electron–hole pairs was assumed. The absorbed photon density per second has a peak value of 8 × 10^23^ cm^−3^·s^−1^, corresponding to the laser pumping density of about 20 W·cm^−2^, with assumed film thickness of 0.2 μm. For the case of n-type InAs thin film used in simulation, the typical drift–diffusion transport framework along with the Shockley–Read–Hall recombination model, Auger recombination model, and the constant mobility model was considered in the whole device structure including the source region. The material parameters are shown below: net doping $$N_{D}$$ 10^17^ cm^−3^, minority carrier lifetime $$\tau$$ 660 ps^6^, electron mobility $$\mu_{n}$$ 4000 cm^2^·V^−1^·s^−1^^21^, hole mobility $$\mu_{p}$$ 60 cm^2^·V^−1^·s^−1^^22^, and Auger coefficient of 2.2 × 10^−27^ cm^6^·s^−1^^23^. The bias of 0.01 V is set on the anode of the device, which corresponds to an applied electric field of 20 V·cm^−1^. These parameters were used throughout this study unless otherwise specified.

In Fig. 1c, we show that the scanning photocurrent profile in the case of 0.2 μm width should exhibit carrier transport properties ideally very close to that of a 1D transport structure, while the 1D results are from the simulator developed previously^6^. Originally, one would expect that with increasing width in 2D transport structures, scanning photocurrent profiles will gradually deviate from that of the ideal 1D transport structure. However, the simulation results in Fig. 1d surprisingly showed almost identical scanning photocurrent profiles from structures with different widths, which indicated the scanning photocurrent profiles independent of the thin film width.

A 1D analytical model for minority carrier decay length extraction from scanning photocurrent profiles in ohmic-contact nanowire devices has been established and confirmed by numerical simulations and experiments^6^. It is known that the minority carrier (hole) concentration decays exponentially with distance away from the generation source in steady state^24^, i.e.

1 $$ \Delta p\left( x \right) = \overline{\Delta p} e^{{ - \left( {x - x_{pump} } \right)/L_{p} }} $$

where $\Delta p$ is the photo-induced hole concentration, $\overline{\Delta p}$ is the minority carrier concentration at excitation, and $L_{p}$ corresponds to the decay length. Minority carrier (hole) decay length for the cathode region $L_{p}$, which is composed of hole drift length $L_{drift,p}$ and hole diffusion length $L_{diff,p}$, can be written as^25^

2 $$ L_{p } = \frac{1}{2}\left[ {L_{drift,p} + \sqrt {L_{drift,p}^{2} + 4L_{diff,p}^{2} } } \right] $$

where $L_{drift,p} = \mu_{p} E_{0} \tau$ and $L_{diff,p} = \sqrt {D_{p} \tau }$. $E_{0}$ is the applied electric field and $D_{p}$ is the hole diffusion coefficient. According to our 1D analytical model^6^, the photocurrent profile on the cathode side can be fitted by the formula

3 $$ a - be^{{x/L_{fit} }} $$

where the symbols $a$ and $b$ are the fitting parameters. And the fitted decay length $L_{fit}$ corresponds to minority carrier (hole) decay length for cathode region $L_{p}$, which must be a positive number.

The calculated decay length $L_{cal}$, *i. e.*, $L_{p}$ obtained from Eq. (2) is 0.324 μm in our case. Based on the results shown in Fig. 1c, it is believed that the fitting method used in scanning photocurrent profiles is valid in the 0.2 μm width case, which still can be considered as a 1D transport structure. Fitted decay length of 0.2 μm-wide thin film extracted in Fig. 1d is around 0.325 μm. Almost identical scanning photocurrent profiles of different thin film widths in Fig. 1d indicate the potential application of Eq. (3) in 2D transport structures.

**Figure 2:**
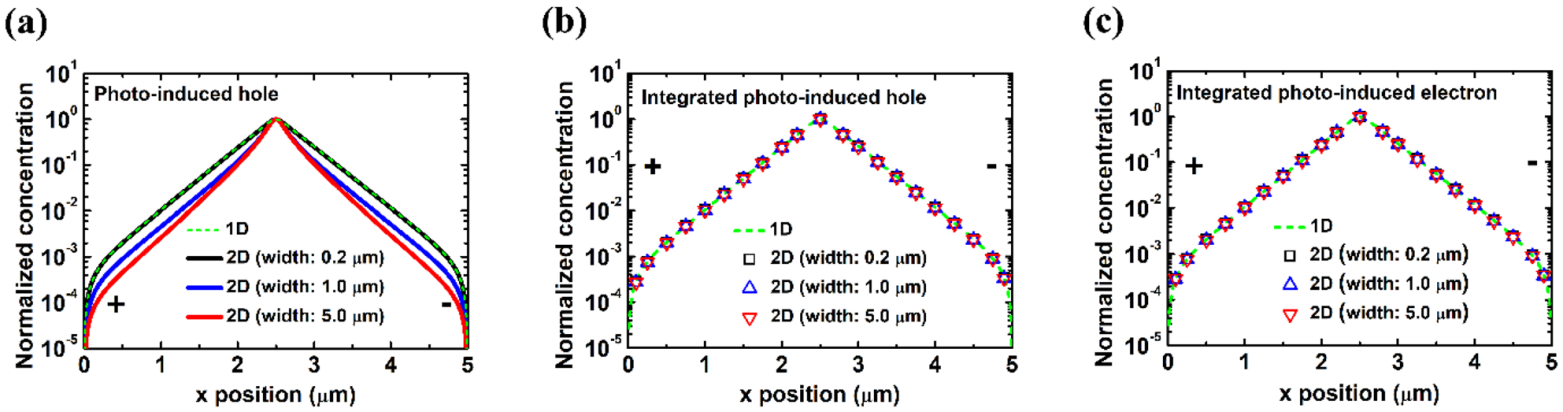
Excitation position is fixed at 2.5 μm. (**a**) Photo-induced hole distribution along $y = 0$ μm. *y*-direction-integrated photo-induced (**b**) hole and (**c**) electron distribution along the channel. The green dashed line represents to the photo-induced carrier distribution along channel in the 1D transport structure, which we used our previous 1D simulator in calculation. Fitted decay length extracted from (**b**) is around 0.325 μm.

We simulated photo-induced carrier concentrations $\Delta n$ and $\Delta p$ in the 2D transport structures, as shown in Fig. 2. 1D carrier distribution using our previous 1D simulator^6^ is also plotted for comparison. Figure 2a is the photo-induced minority carrier (hole) spatial distribution along $y = 0$ μm with three different widths, while the excitation position is fixed at $x = 2.5$ μm. It is expected that the hole distribution along the channel is different between these three widths due to the nature of 2D carrier transport. Furthermore, a simple-exponential-decay function with decay length as the fitting parameter cannot adequately describe the carrier distribution in 2D transport structures.

With the simulation results of $\Delta n$ and $\Delta p$, we can calculate the *y*-direction-integrated photo-induced carrier distribution along the channel in $x$ direction for three devices of different widths, which are shown in Fig. 2b and c. As Fig. 2b and c show, the *y*-direction-integrated photo-induced carrier distribution along the channel can be well fitted with a simple-exponential-decay function and is almost independent of the width. It is also almost identical to that of the 1D transport structure. It should be noted that the electron decay length extracted from Fig. 2c is almost identical to that of the minority carrier (hole) in Fig. 2b. This is due to the screening effect^24^ and what we determine from scanning photocurrent profiles is the minority carrier decay length^6^. According to the simulation results in Fig. 2b and c, a simple-exponential-decay function with the decay length as the fitting parameter can adequately describe the *y*-direction-integrated carrier distribution in 2D transport structures with finite width, and its decay length corresponds to $L_{p}$ from Eq. (2).

With the numerically obtained carrier distribution on *x–y* plane, the photocurrent density can be observed analytically now since it can be decomposed into the drift current and the diffusion current contributed by both carriers. It should be noted that electrodes cover the whole terminal on both sides and collect the current only in $x$ direction. Therefore, the photocurrent density in $x$ direction $\Delta J_{x}$ under weak optical excitation^6^ can be expressed in the following

4 $$ \frac{{\Delta J_{x} }}{q} \approx \mu_{n} \left( {E_{0} \Delta n + \Delta E_{x} n_{0} } \right) + D_{n} \frac{\partial \Delta n}{{\partial x}} + \mu_{p} E_{0} \Delta p - D_{p} \frac{\partial \Delta p}{{\partial x}} $$

where $n_{0}$ and $p_{0}$ are carrier concentrations without excitation, $\Delta E_{x}$ is the photo-induced electric field in $x$ direction, and $D_{n}$ is the electron diffusion coefficient, while $E_{0}$ is uniform along the channel in $x$ direction. In the 2D transport structure, the total photocurrent at the electrodes can be obtained by integrating $\Delta J_{x}$ in the $y$ direction

5 $$ \begin{aligned} \mathop \smallint \limits_{{\frac{ - w}{2}}}^{\frac{w}{2}} \frac{{\Delta J_{x} }}{q}\, dy & \approx \mathop \smallint \limits_{{\frac{ - w}{2}}}^{\frac{w}{2}} \mu_{n} \left( {E_{0} \Delta n + \Delta E_{x} n_{0} } \right)\, dy + \mathop \smallint \limits_{{\frac{ - w}{2}}}^{\frac{w}{2}} D_{n} \frac{\partial \Delta n}{{\partial x}}\, dy + \mathop \smallint \limits_{{\frac{ - w}{2}}}^{\frac{w}{2}} \mu_{p} E_{0} \Delta p\, dy - \mathop \smallint \limits_{{\frac{ - w}{2}}}^{\frac{w}{2}} D_{p} \frac{\partial \Delta p}{{\partial x}}\, dy \\ & = \mu_{n} E_{0} \mathop \smallint \limits_{{\frac{ - w}{2}}}^{\frac{w}{2}} \Delta n\, dy + \mu_{n} n_{0} \mathop \smallint \limits_{{\frac{ - w}{2}}}^{\frac{w}{2}} \Delta E_{x} \, dy + D_{n} \frac{d}{dx}\left( {\mathop \smallint \limits_{{\frac{ - w}{2}}}^{\frac{w}{2}} \Delta n\, dy} \right) + \mu_{p} E_{0} \mathop \smallint \limits_{{\frac{ - w}{2}}}^{\frac{w}{2}} \Delta p\, dy - D_{p} \frac{d}{dx}\left( {\mathop \smallint \limits_{{\frac{ - w}{2}}}^{\frac{w}{2}} \Delta p\, dy} \right) \\ \end{aligned} $$

where symbol ${\text{w}}$ is the width in ${\text{y}}$ direction. It should be noted that except for $\Delta n$, $\Delta p$, and $\Delta E_{x}$, other parameters are assumed to be constant in $y$ direction and therefore can be moved out of the integration. As Eq. (5) shows, it still exhibits the same form as in our 1D analytical model.

*y*-direction-integrated photo-induced carrier distributions results in almost the same normalized carrier distribution along the channel from thin-film structures with different widths, as shown in Fig. 2b and c. The *y*-direction-integrated photo-induced electric field in $x$ direction $\mathop \int \nolimits_{{\frac{ - w}{2}}}^{\frac{w}{2}} \Delta E_{x} dy$ is also verified numerically to be independent of the width (see Supplementary Information S1). After inserted into Eq. (5), the *y*-direction-integrated photo-induced carrier distribution/electric field will lead to the scanning photocurrent profiles almost independent of the width, as already demonstrated in Fig. 1d by simulation directly.

**Figure 3:**
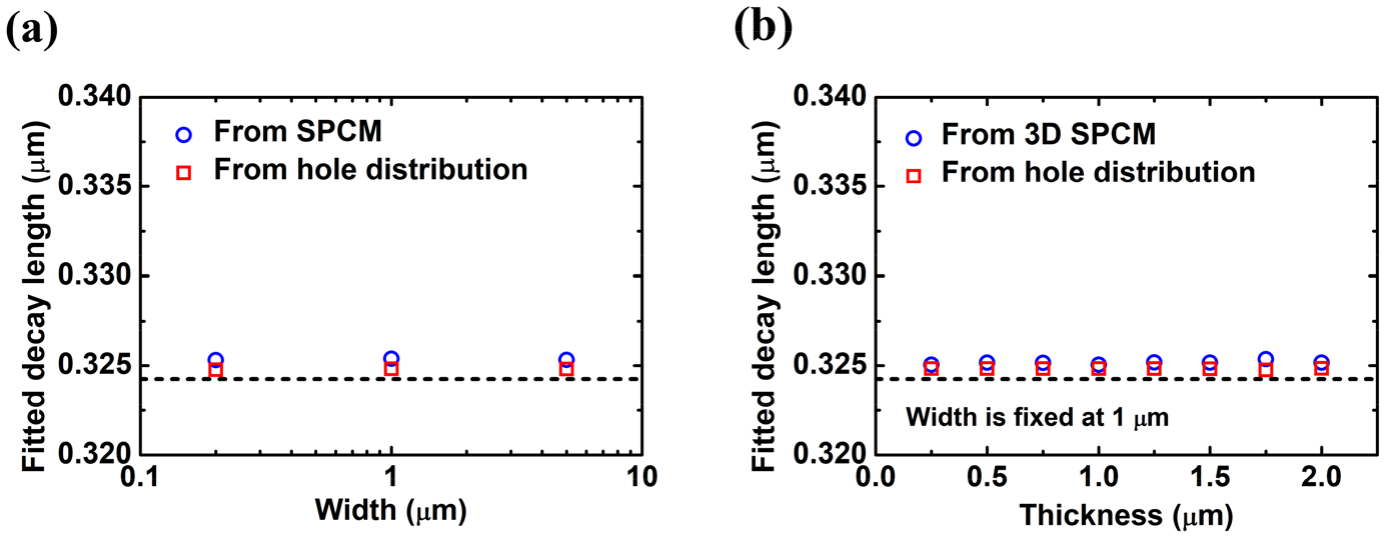
Fitted decay lengths extracted from scanning photocurrent profiles and from integrated photo-induced hole distributions (**a**) in 2D transport structures with different width and (**b**) in 3D transport structures with different thickness while the width is fixed at 1 μm. Calculated decay length obtained from Eq. (2) is shown as the dashed line.

$L_{fit}$ extracted from scanning photocurrent profiles and the decay length extracted from the *y*-direction-integrated photo-induced carrier distribution are depicted in Fig. 3a with different width. The deviation of both fitted decay lengths from the calculated decay length $L_{cal}$ (0.324 μm) is less than 0.5%. They also show negligible dependence on width. According to the assumption of electrodes covering the whole terminal on both sides, we can also express Eq. (5) in the three-dimensional (3D) transport structure (see Supplementary Information). We built a 3D structure in TCAD simulations and set the width to be fixed at 1 μm. The maximum thickness we considered is 2 μm, which is much larger than the calculated decay length. Both the scanning photocurrent profile and the *y*- and *z*-direction-integrated photo-induced carrier distribution along the channel in $x$ direction are almost independent of the thickness. The verification of the fitted decay length with different thicknesses is shown in Fig. 3b. It should be noted that the scanning photocurrent profile and the *y*- and *z*-direction-integrated photo-induced carrier distribution in a 3D transport structure with electrodes covering the whole terminal on both sides will reduce to those of the 1D transport structure, appropriately described by the 1D model. Based on the verification in Fig. 3, although carrier decay length may not be adequately defined in both 2D and 3D transport structures where the carrier distributions are not simple-exponential-decay functions, $L_{fit}$ from the scanning photocurrent profile can still be used to obtain the minority carrier mobility if lifetime is known.

**Figure 4:**
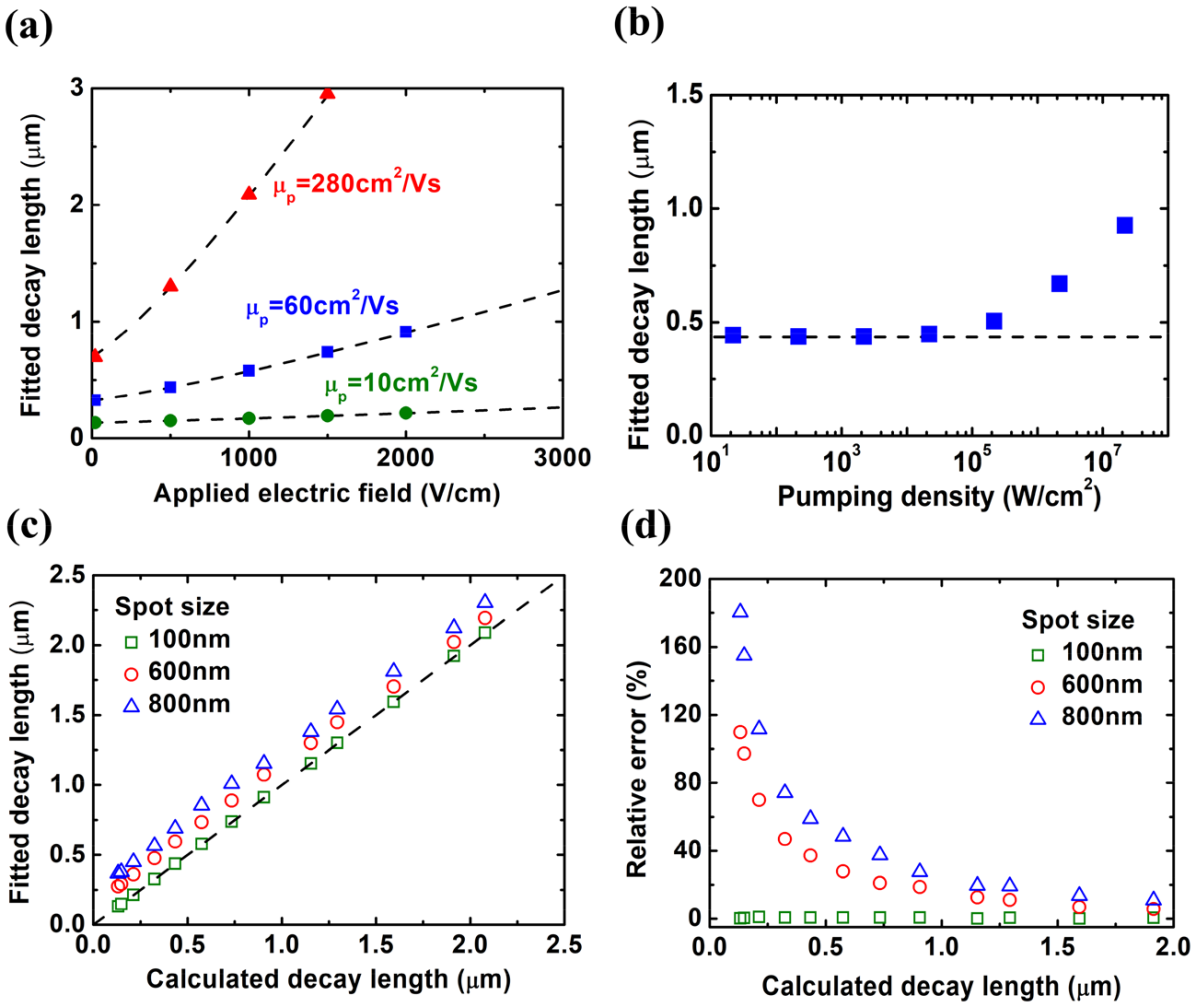
(**a**, **b**) Fitted decay length extracted from scanning photocurrent profiles with varied (**a**) applied electric field and (**b**) pumping density. The dashed line corresponds to the calculated decay length obtained from Eq. (2). (**c**) The relations between fitted decay length and calculated decay length with varied laser spot size. The $x = {\text{y}}$ line is shown as the dashed line for clarity. (**d**) The relative error calculated in (**c**) with varied laser spot size.

After confirming the feasibility of the 1D analytical model in both 2D and 3D transport structures, we would like to explore the appropriate operation conditions of the 1D analytical model using the less time-consuming 2D-structured model instead of 3D-structured model in simulation for 2D transport structures. Figure 4a shows $L_{fit}$ from scanning photocurrent profiles with the varied applied electric field across the channel, and the dashed line represents $L_{cal}$ obtained from Eq. (2). $L_{fit}$ is in agreement with $L_{cal}$ for all cases in that figure, where the hole mobility varying from 10 to 280 cm^2^·V^−1^·S^−1^^22^. In Fig. 4b, $L_{fit}$ from scanning photocurrent profiles is plotted against the pumping density with the applied electric field of 500 V·cm^−1^ and the hole mobility of 60 cm^2^·V^−1^·S^−1^. We found that the corresponding $L_{fit}$ remains almost unchanged when the pumping density is below 2 × 10^4^ W·cm^−2^ and its value is consistent with $L_{cal}$ (0.434 μm), represented by the dashed line. The fitting fails for stronger pumping due to the invalid assumption of the analytical model $n_{0}$ $\gg$ $\Delta n$, $\Delta p$ under this condition^6^. For the laser spot size larger than or equal to 100 nm, the relations between $L_{fit}$ and $L_{cal}$ with three spot sizes are shown in Fig. 4c. And the relative error, defined as $\left| {L_{fit} - L_{cal} } \right|/L_{cal} \times 100\%$ is shown in Fig. 4d. The fitting error with increasing spot size is due to the deviation to the assumption of an ideal delta-function source. It should be noticed that $L_{fit}$ and $L_{cal}$ are in one-to-one correspondence with chosen spot sizes, so the relative error caused by the finite laser spot can be eliminated if we know the actual spot size. More details about the origin of the relative error and the method to reduce it in those operation conditions can be seen in our previous report^6^.

After studying 2D transport structures with ohmic contact, we also observed the scanning photocurrent profiles in Schottky-contact InAs thin-film devices. As already shown in p-n junction^3,4^ and Schottky-contact^7^ nanowire devices, both photo-induced electrons and holes can diffuse in the charge neutral region with a simple-exponential-decay distribution and be separated by the built-in electric field of the space charge region. On the other hand, total current is dominated by the minority carrier (hole) diffusion current at the edge of the depletion region where majority carrier (electron) currents cancel with each other. That leads to the simple-exponential-decay scanning photocurrent profile^7^. Due to the charge screening effect, both majority and minority carriers have the same decay length, and what we determine from scanning photocurrent profiles is the minority carrier decay length.

**Figure 5:**
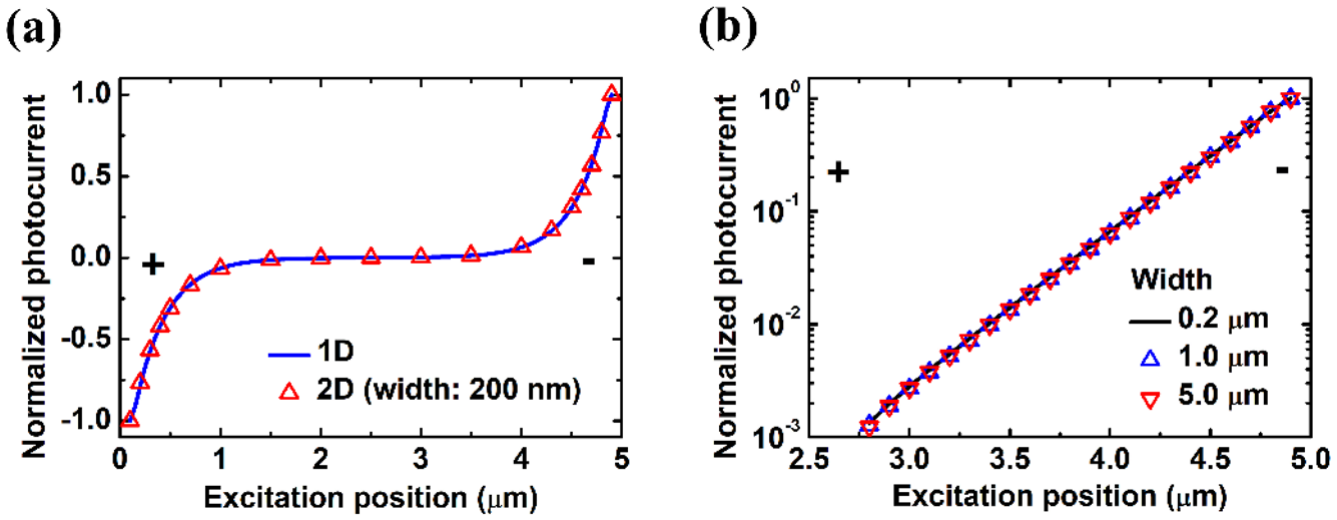
In Schottky-contact based devices. (**a**) Scanning photocurrent profiles of the 0.2 μm wide case compared with the 1D transport structure. Our previous simulator was used in 1D simulation. (**b**) Scanning photocurrent profiles on the cathode side with different widths. Fitted decay length extracted from (**b**) is around 0.323 μm.

Apart from the discussion about 1D transport structures above, we would like to explore the SPCM in 2D transport structures with TCAD simulation now. We used the same material parameters as before but with Schottky contact of 0.055 eV barrier height^26^ for simulations. Zero bias was applied in these cases. In Fig. 5a, the scanning photocurrent profile in the case of 0.2 μm device width is compared with that of the 1D transport structure where our previous simulator was used^6^. The profile in the 0.2 μm-wide transport structure is ideally very close to that of the 1D transport structure and both of them can be well fitted with a simple-exponential-decay function near the contact regions. The extracted minority carrier diffusion length $L_{fit}$ is 0.323 μm while the calculated diffusion length $L_{diff,p} = \sqrt {D_{p} \tau }$ is 0.320 μm. They are very consistent with each other. In Fig. 5b, almost identical scanning photocurrent profiles of different device widths is observed on the cathode side under zero bias condition. It should be noted that the decay length as the fitting parameter of scanning photocurrent profiles can also adequately describe the *y*-direction-integrated carrier distribution. For Schottky-contact-based structures, we also verified that the scanning photocurrent and the *y*- and *z*-direction-integrated photo-induced carrier distribution profile in a 3D transport structure with electrodes covering the whole terminal on both sides will reduce to those of the 1D transport structure (see Supplementary Information Fig. S2). Therefore, we have confirmed the validity of a simple-exponential-decay function fitted in scanning photocurrent profiles of Schottky-contact 2D transport structures. And these simulation results are in agreement with the experimental reports in the literature^9,10^.

**Figure 6:**
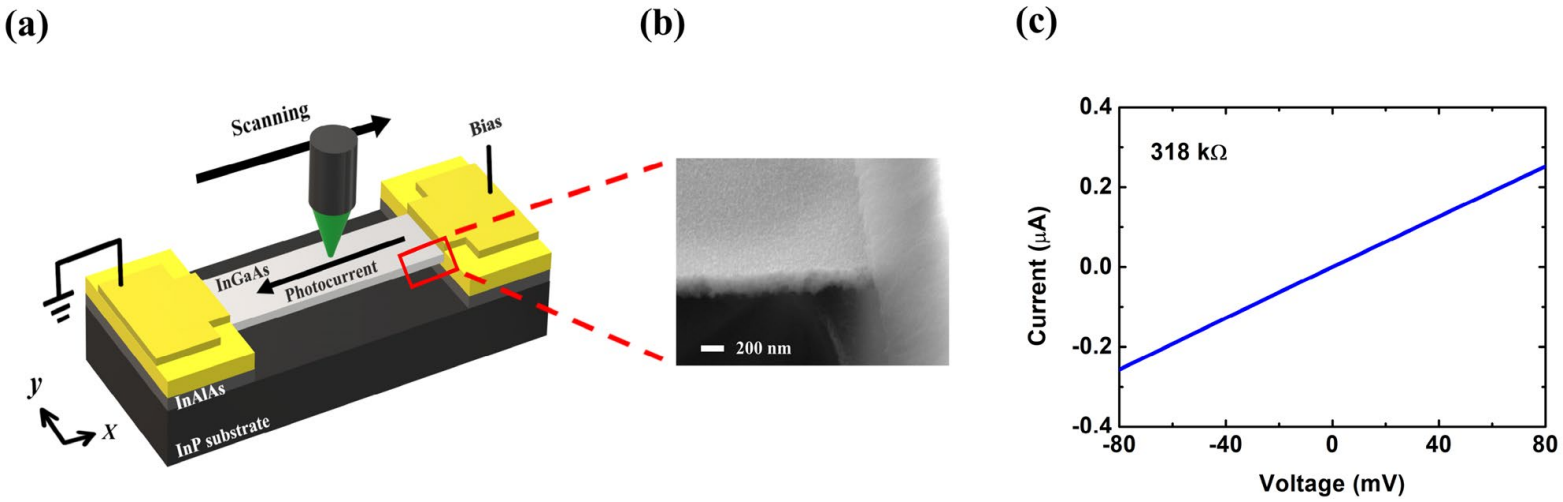
(**a**) Schematic of the SPCM setup in an InGaAs air-bridge two-terminal thin-film device. (**b**) SEM images for one side of the measured InGaAs air-bridge two-terminal thin-film device. (**c**) I–V characteristics. $L_{ch}$ of the measured thin-film device is 15 μm, and the width is 5 μm. *(**a**) is created using Microsoft Paint 3D ver. 6.2105.4017.0-URL: https://www.microsoft.com/en-us/p/paint-3d/9nblggh5fv99?cid=msft_web_chart.

Finally, we demonstrated the experimental SPCM from a p-type InGaAs air-bridge two-terminal thin-film device with 15 μm in length between electrodes $L_{ch}$ and 5 μm in width (see the Methods for details of the device fabrication). Figure 6a shows the Schematic of the SPCM setup with this InGaAs device. A scanning electron microscopy (SEM) image from the side view is shown in Fig. 6b. Figure 6c shows the linear current–voltage (I-V) curve of this device, which indicates an ohmic-contact feature. The resistance of the device can be extracted to be 318 kΩ.

**Figure 7:**
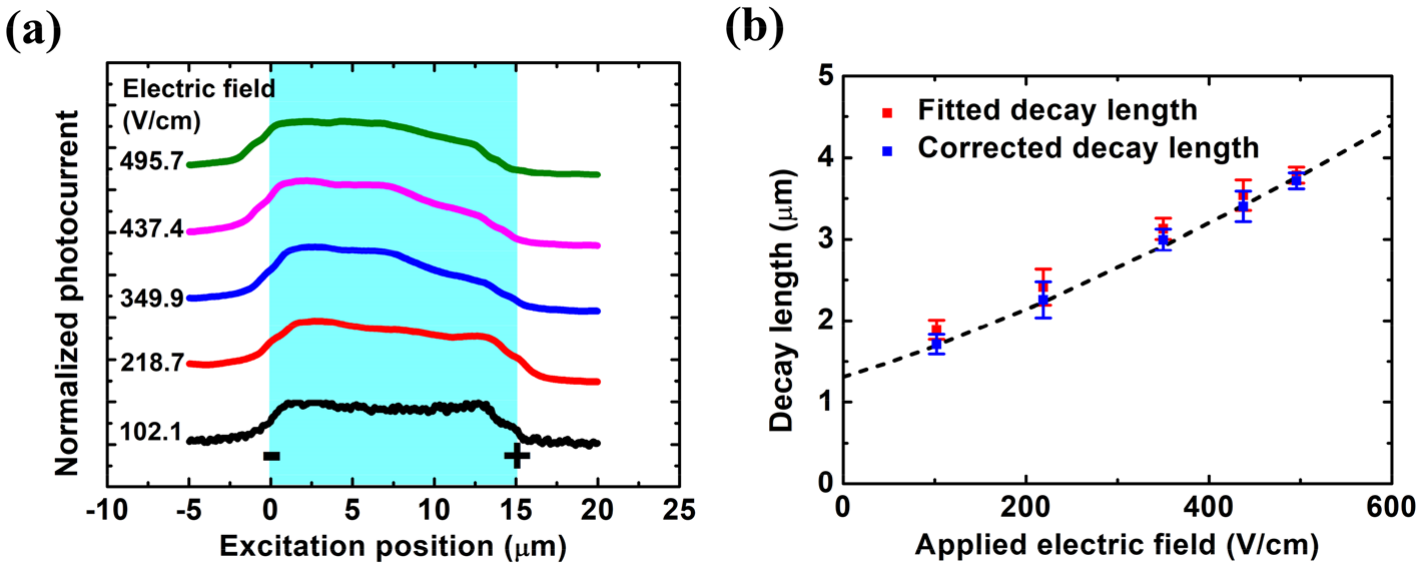
SPCM measurement in an InGaAs air-bridge two-terminal thin-film device. (**a**) Scanning photocurrent profiles with varied applied electric field under pumping density of 29 W·cm^−2^. (**b**) $L_{fit}$ and corrected decay lengths fitted in (**a**) as a function of the applied electric field. The dashed line shows the fitting curve using Eq. (2). $L_{ch}$ of the measured thin-film device is 15 μm shown with blue background and the excitation spot size is around 800 nm. The photocurrent profiles in (**a**) is offset vertically for clarity.

Scanning positions of experimental measurements (see the Methods for details of the SPCM) along the device are the same as we described in Fig. 1b, except the cathode is at $$x = 0$$ μm and the anode is at $$ x = L_{ch}$$. Scanning photocurrent profiles with varied applied electric field under pumping density of 29 W·cm^−2^ is shown in Fig. 7a. $$L_{fit}$$ and corrected decay lengths (see Supplementary Information Fig. S3) as a function of the applied electric field are shown in Fig. 7b. It is noted that $$L_{fit}$$ should be fitted on the anode side of photocurrent profiles because of the stronger charge screening effect on that side of our p-type InGaAs device^6,24,27^, which leads to the opposite fitting direction in photocurrent profiles compared with that of the previous 1D analytical model for n-type nanowire devices^6^ (see Supplementary Information). It is in agreement with both our previous study^6^ and the discussion in this paper that scanning photocurrent profiles under appropriate weak excitation in Fig. 7a can be fitted by Eq. (3), instead of a simple-exponential-decay function. By applying Eq. (2) with the corrected decay length together with the application of Einstein relation as the dashed line in Fig. 7b, the mobility-lifetime product is extracted to be 6.6 × 10^–7^ cm^2^·V^−1^ and the corresponding electron diffusion length is 1.3 μm. The obtained electron diffusion length is about 35% lower than that measured and estimated in experimental results of InGaAs p-n junctions^28^. The thicker structure (8 μm) in that InGaAs p-n junctions compared with ours (200 nm) may contribute to the difference of mobility-lifetime product between these two experimental results.

## Conclusion

In this paper, we studied carrier transport properties in 2D transport structures with ohmic or Schottky contact. Firstly, the collected photocurrent in two-terminal InAs thin-film devices with electrodes covering the whole terminal on both sides were simulated. Originally, one would expect that with increasing widths in 2D transport structures, scanning photocurrent profiles will gradually deviate from that of the ideal 1D transport structures. However, the simulation results surprisingly showed almost identical scanning photocurrent profiles from structures with different widths.

Then, we showed that the fitting formula from our previous 1D analytical model can be used to extract minority carrier decay length in such ohmic-contact 2D transport structures. The scanning photocurrent profile and the y-direction-integrated minority carrier distribution do not share the same functional form, but both have the same fitting parameter- minority carrier decay length. The feasibility study of this fitting method with different operation conditions was also presented. We also justified the application of a simple-exponential-decay function for minority carrier diffusion length extraction from scanning photocurrent profiles of Schottky-contact 2D transport structures. Furthermore, our simulation results indicated that scanning photocurrent profiles in the ohmic- or Schottky-contact 3D transport structures with electrodes covering the whole terminal on both sides will reduce to those described by the corresponding 1D fitting formulae, respectively. Finally, the experimental SPCM was applied on a p-type InGaAs air-bridge two-terminal thin-film device with ohmic contact. The SPCM data with varied applied electric field was recorded and analyzed with our previous 1D analytical model and the electron mobility-lifetime product can be extracted.

According to the numerical and experimental results above, different fitting formulae for ohmic- or Schottky-contact SPCM 1D devices can be further used in the corresponding 2D transport structures with finite width. This study opens up a new opportunity to extract the minority carrier decay length and to obtain the mobility-lifetime product of 2D transport structures.

## Methods

### Device fabrication

The sample was first grown by molecular beam epitaxy (MBE) on a (100) InP semi-insulating substrate. A 50 nm thick In_0.52_Al_0.48_As layer followed by a 200 nm thick Be-doped (10^17^ cm^−3^) In_0.53_Ga_0.47_As layer with a 30 nm thick Be-doped (2 × 10^19^ cm^−3^) In_0.53_Ga_0.47_As contact layer on top. The thin-film device channel was patterned by 1st UV lithography with 5 μm in width and 17 μm in length. Then Inductively coupled plasma-reactive ion etching (ICP-RIE) was used and the etching depth is about 360 nm to make sure the entire In_0.53_Ga_0.47_As thin film channel was formed. To ensure the good ohmic contact between metal and thin films, 2^nd^ UV lithography patterned Pt (9 nm)/Ti (15 nm)/Pt (15 nm)/Au (100 nm) film^29^ was deposited by electron-beam evaporation and then followed by a lift-off process. The defined channel length between electrodes is 15 μm. Finally, the top 30 nm thick In_0.53_Ga_0.47_As contact layer and the 50 nm thick In_0.52_Al_0.48_As layer were removed using H_2_SO_4_-based^30^ and HCl-based^31^ solutions, respectively, to form a uniformly-doped thin film structure for 2D transport study. It should be noted that the developed SPCM fitting formula using the 3D transport model is the same as that with the 2D transport structure. Therefore, the assumption of 2D carrier transport in the 200 nm thick thin film device is valid.

### Scanning photocurrent microscopy

A continuous-wave laser with wavelength of 532 nm passed through the chopper and was focused onto an InGaAs thin-film device by a 100× objective lens with finite excitation spot size of about 800 nm. The excitation position was precisely controlled using a piezo-electric stage. A source meter unit was used to apply bias across the thin-film device and the photocurrent was measured with a lock-in amplifier.

## Supplementary Information

Supplementary Information.

## Data Availability

The data reported in this paper are available upon request.
