# Supplementary material for: Minority carrier decay length extraction from scanning photocurrent profiles in two-dimensional carrier transport structures
Source: Sci Rep. 2021 Nov 8;11:21863. doi: 10.1038/s41598-021-01446-5 (PMC8575939; doi:10.1038/s41598-021-01446-5)
Supplement: Supplementary file 1 — Supplementary Information. [file 41598_2021_1446_MOESM1_ESM.pdf]

Supplementary Information

# Minority Carrier Decay Length Extraction from Scanning Photocurrent Profiles in Two-Dimensional Carrier Transport Structures


**Yu-Chien Wei,[1] Cheng-Hao Chu,[1] and Ming-Hua Mao [1,2,3*]**

[1]Graduate Institute of Electronics Engineering, National Taiwan University, No. 1, Roosevelt Rd. Sec. 4, Taipei 10617, Taiwan

[2]Department of Electrical Engineering, National Taiwan University, No. 1, Roosevelt Rd. Sec. 4, Taipei 10617, Taiwan

[3]Graduate Institute of Photonics and Optoelectronics, National Taiwan University, No. 1, Roosevelt Rd. Sec. 4, Taipei 10617, Taiwan

*Correspondence and requests for materials should be addressed to M.-H.M. (email: mhmao@ntu.edu.tw)


**Verification of the *y*-direction-integrated photo-induced electric field in a 2D transport structure with ohmic contact**

We built a 2D structure of InAs with ohmic contact in TCAD simulations. The channel length $L_{ch}$ and the width of this two-terminal thin-film device are both 5 μm. It should be noted that electrodes cover the whole terminal of the structure on both sides. For the case of n-type InAs thin film used in simulation, the typical drift-diffusion transport framework along with the Shockley-Read-Hall recombination model, Auger recombination model, and the constant mobility model was considered. The material parameters are shown below: net doping $N_D$ $10^{17}$ $cm^{-3}$, minority carrier lifetime $\tau$ 660 ps [1], electron mobility $\mu_n$ 4000 $cm^2 \cdot V^{-1} \cdot s^{-1}$ [2], hole mobility $\mu_p$ 60 $cm^2 \cdot V^{-1} \cdot s^{-1}$ [3], Auger coefficient of $2.2 \times 10^{-27}$ $cm^6 \cdot s^{-1}$ [4]. The bias of 0.01 V is set on the anode of the device, which corresponds to an applied electric field of 20 $V \cdot cm^{-1}$. Figure S1 is the *y*-direction-integrated photo-induced electric field in $x$ direction along the channel, while the excitation position is at the center of the channel with the corresponding pumping density of about 20 $W \cdot cm^{-2}$. With varied channel width from 200 to 5000 nm, the y-direction-integrated photo-induced electric field remains almost the same.

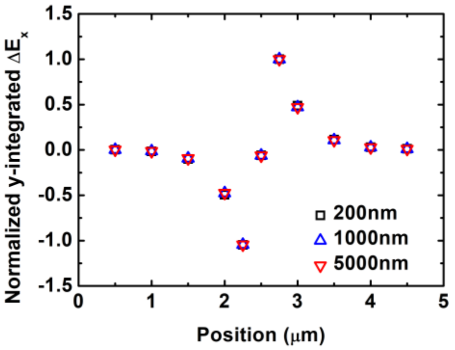


**Figure S1.** *y*-direction-integrated photo-induced electric field in $x$ direction along the channel.

**Expression of the total photocurrent in a 3D transport structure**

The expression of total photocurrent in x direction $\Delta J_x$ under weak optical excitation for a 2D transport structure with electrodes covering the whole terminal of the thin film on both sides can be written as

$$\int_{-\frac{w}{2}}^{\frac{w}{2}} \frac{\Delta J_x}{q} dy \approx \int_{-\frac{w}{2}}^{\frac{w}{2}} \mu_n (E_0 \Delta n + \Delta E_x n_0)\, dy + \int_{-\frac{w}{2}}^{\frac{w}{2}} D_n \frac{\partial \Delta n}{\partial x} dy + \int_{-\frac{w}{2}}^{\frac{w}{2}} \mu_p E_0 \Delta p\, dy - \int_{-\frac{w}{2}}^{\frac{w}{2}} D_p \frac{\partial \Delta p}{\partial x} dy$$

$$= \mu_n E_0 \int_{-\frac{w}{2}}^{\frac{w}{2}} \Delta n\, dy + \mu_n n_0 \int_{-\frac{w}{2}}^{\frac{w}{2}} \Delta E_x\, dy + D_n \frac{d}{dx}\left(\int_{-\frac{w}{2}}^{\frac{w}{2}} \Delta n\, dy\right) + \mu_p E_0 \int_{-\frac{w}{2}}^{\frac{w}{2}} \Delta p\, dy - D_p \frac{d}{dx}\left(\int_{-\frac{w}{2}}^{\frac{w}{2}} \Delta p\, dy\right) \quad \text{(S1)}$$

where $n_0$, $p_0$ are the carrier concentration without excitation and $\Delta n$, $\Delta p$ are their optical-excitation-induced changes. $E_0$ is the applied electric field and $\Delta E_x$ is the photo-induced electric field in $x$ direction. $\mu_{n,p}$ and $D_{n,p}$ are the electron/hole mobilities and diffusion coefficients, respectively. Symbol w is the width in y direction. It should be noted that except for $\Delta n$, $\Delta p$, and $\Delta E_x$, other parameters are assumed to be constant in $y$ direction and therefore can be moved out of the integration. We can also express the total photocurrent at the electrodes in 3D transport structure with electrodes covering the whole terminal of the structure on both sides

$$\int_{-\frac{T}{2}}^{\frac{T}{2}} \int_{-\frac{w}{2}}^{\frac{w}{2}} \frac{\Delta J_x}{q} dydz \approx \int_{-\frac{T}{2}}^{\frac{T}{2}} \int_{-\frac{w}{2}}^{\frac{w}{2}} \mu_n (E_0 \Delta n + \Delta E_x n_0)\, dydz + \int_{-\frac{T}{2}}^{\frac{T}{2}} \int_{-\frac{w}{2}}^{\frac{w}{2}} D_n \frac{\partial \Delta n}{\partial x} dydz + \int_{-\frac{T}{2}}^{\frac{T}{2}} \int_{-\frac{w}{2}}^{\frac{w}{2}} \mu_p E_0 \Delta p\, dydz - \int_{-\frac{T}{2}}^{\frac{T}{2}} \int_{-\frac{w}{2}}^{\frac{w}{2}} D_p \frac{\partial \Delta p}{\partial x} dydz$$

$$= \mu_n E_0 \int_{-\frac{T}{2}}^{\frac{T}{2}} \int_{-\frac{w}{2}}^{\frac{w}{2}} \Delta n\, dydz + \mu_n n_0 \int_{-\frac{T}{2}}^{\frac{T}{2}} \int_{-\frac{w}{2}}^{\frac{w}{2}} \Delta E_x\, dydz + D_n \frac{d}{dx}\left(\int_{-\frac{T}{2}}^{\frac{T}{2}} \int_{-\frac{w}{2}}^{\frac{w}{2}} \Delta n\, dydz\right) + \mu_p E_0 \int_{-\frac{T}{2}}^{\frac{T}{2}} \int_{-\frac{w}{2}}^{\frac{w}{2}} \Delta p\, dydz - D_p \frac{d}{dx}\left(\int_{-\frac{T}{2}}^{\frac{T}{2}} \int_{-\frac{w}{2}}^{\frac{w}{2}} \Delta p\, dydz\right) \quad \text{(S2)}$$

where symbol T is the thickness in z direction. It should be also noted that except for $\Delta n$, $\Delta p$, and $\Delta E_x$, other parameters are assumed to be constant in $y$ and $z$ direction and therefore they can be moved out of the integration. As equation (S2) shows, it still exhibits the same form as in our 1D analytical model[1].

**Verification of the *y*- and *z*-direction-integrated photo-induced carrier distribution and scanning photocurrent profiles in a 3D transport structure with Schottky contact**

We built a 3D structure of InAs with Schottky contact in TCAD simulations and set the width to be fixed at 1 μm. The maximum thickness we considered is 2 μm, which is much larger than the calculated decay length introduced below. It should be noted that electrodes cover the whole terminal of the structure on both sides. For the case of n-type InAs thin film used in simulation, the typical drift-diffusion transport framework along with the Shockley-Read-Hall recombination model, Auger recombination model, and the constant mobility model was considered. The material parameters are shown below: net doping $N_D$ $10^{17}$ $cm^{-3}$, minority carrier lifetime $\tau$ 660 ps [1], electron mobility $\mu_n$ 4000 $cm^2 \cdot V^{-1} \cdot s^{-1}$ [2], hole mobility $\mu_p$ 60 $cm^2 \cdot V^{-1} \cdot s^{-1}$ [3], Auger coefficient of $2.2\times10^{-27}$ $cm^6 \cdot s^{-1}$ [4], and the Schottky contact with barrier of 0.055 eV [5]. Zero bias was applied in these cases. In Fig. S2, both the *y*- and *z*-direction-integrated photo-induced hole distribution along the channel in x direction and the scanning photocurrent profile are almost independent of the thickness. It shows that the scanning photocurrent profile and the *y*- and *z*-direction-integrated photo-induced carrier distribution in a 3D transport structure with electrodes covering the whole terminal on both sides will reduce to those of the 1D transport structure,

appropriately described by the 1D simple-exponential-decay function. The extracted decay lengths from both profiles in Fig. S2(a) and S2(b) are around 0.321 μm.

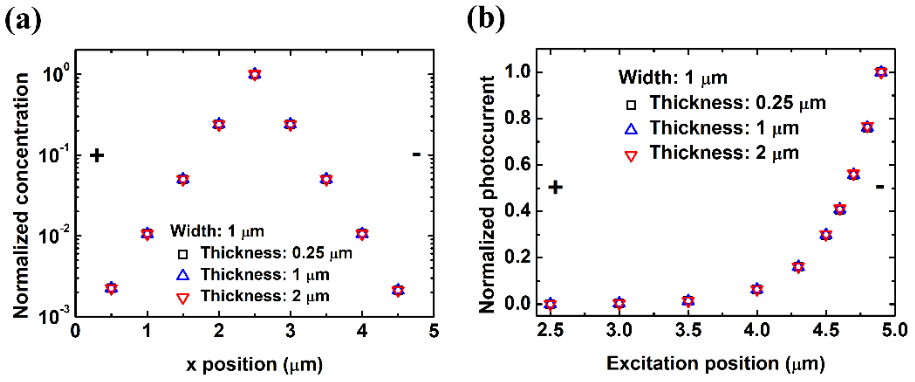


**Figure S2.** In Schottky-contact based devices. (a) *y*- and *z*-direction-integrated photo-induced hole distribution along the channel with excitation position of $x = 2.5$ μm. (b) Scanning photocurrent profiles on the cathode side.

**Simulation results of the relations between fitted decay length and calculated decay length with laser spot size of 800 nm in InGaAs 2D transport structure with ohmic contact**

We built a 2D structure of InGaAs in TCAD simulations with 15 μm in length between electrodes $L_{ch}$ and 5 μm in width. It should be noted that electrodes cover the whole terminal of the structure on both sides. The typical drift-diffusion transport framework along with the Shockley-Read-Hall recombination model, Auger recombination model and the constant mobility model was considered. Figure S3 shows the relations between fitted decay length $L_{fit}$ and calculated decay length $L_{cal}$ with laser spot size of 800 nm.

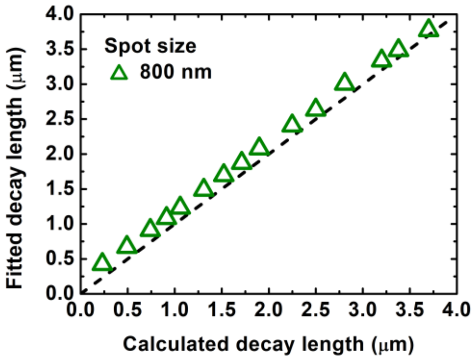


**Figure S3.** The relations between fitted decay length and calculated decay length with laser spot size of 800 nm. The $x = y$ line is shown as the dashed line for clarity.

**1D analytical model for p-type two-terminal devices with ohmic contact**

In the p-type devices, it is known that the minority carrier (electron) concentration decays exponentially with distance away from the generation source in steady state[6], i.e.

$$\Delta n(x) = \overline{\Delta n} e^{-(x - x_{pump})/L_n} \quad \text{(S3)}$$

where $\overline{\Delta n}$ is the minority carrier concentration at excitation, and $L_n$ corresponds to the decay length. Minority carrier (electron) decay length for the anode region $L_n$, which is composed of electron drift length $L_{drift,n}$ and electron diffusion length $L_{diff,n}$, can be written as[7]

$$L_n = \frac{1}{2}\left[L_{drift,n} + \sqrt{L_{drift,n}^2 + 4L_{diff,n}^2}\right] \quad \text{(S4)}$$

where $L_{drift,n} = \mu_n E_0 \tau$ and $L_{diff,n} = \sqrt{D_n \tau}$. Due to the charge screening effect, the concentration is almost governed by the minority carriers[6,8] and the screening effect becomes stronger with higher minority carrier concentration. As the result, the decay length for holes in the anode region $L_{anode,p}$ will be the same as that for electrons $L_{anode,n}$ with large bias, $L_{anode} = L_{anode,n} = L_{anode,p}$. Therefore, take the derivation of our previous 1D analytical model in n-type devices[1] for reference, the photocurrent profile as a function of the excitation position $x_{pump}$ can be written as

$$\Delta J \approx \frac{q^2 \mu_n n_0}{\varepsilon L_{ch}}\left[-\alpha e^{\frac{(-x_{pump})}{L_{cathode,n}}} - \beta e^{\frac{(-x_{pump})}{L_{cathode,p}}} - \gamma e^{\frac{x_{pump}}{L_{anode}}} + \delta\right] \quad \text{(S5)}$$

where $\varepsilon$ is the dielectric constant, $q$ is the fundamental charge, $L_{ch}$ is the channel length, and $\alpha$, $\beta$, $\gamma$, $\delta$ are constants. The photocurrent profile on the anode side can be fitted by the formula

$$a - be^{x/L_{fit}} \quad \text{(S6)}$$

where the symbols $a$ and $b$ are the fitting parameters. And the fitted decay length $L_{fit}$ corresponds to minority carrier (electron) decay length for anode region $L_n$.

## References


1 Chu, C. H., Mao, M. H., Yang, C. W. & Lin, H. H. A New Analytic Formula for Minority Carrier Decay Length Extraction from Scanning Photocurrent Profiles in Ohmic-Contact Nanowire Devices. *Sci Rep* **9**, 10, doi:10.1038/s41598-019-46020-2 (2019).

2 Takita, H. et al. Electron transport properties of InAs ultrathin films obtained by epitaxial lift-off and van der Waals bonding on flexible substrates. Appl. Phys. Lett. 97, 3, doi:10.1063/1.3459137 (2010).

3 Sotoodeh, M., Khalid, A. H. & Rezazadeh, A. A. Empirical low-field mobility model for III-V compounds applicable in device simulation codes. J. Appl. Phys. 87, 2890-2900, doi:10.1063/1.372274 (2000).

4 Mikhailova, M. P. Levinshtein, M., Rumyantsev, S. & Shur, M. (ed.) *Handbook Series on Semiconductor Parameters*. Chapter 7, 159 (World Scientific, 1996).

5 Feng, B. Y. *et al.* Schottky barrier heights at the interfaces between pure-phase InAs nanowires and metal contacts. *J. Appl. Phys.* **119**, 6, doi:10.1063/1.4941391 (2016).

6 Neamen, D. A. *Semiconductor Physics and Devices: Basic Principles*. 194-203 (McGraw-Hill, 2012).

7 Ferry, D. *Semiconductor Transport*. 277-283 (Taylor & Francis, 2000).

8 Chazalviel, J. N. *Coulomb Screening by Mobile Charges: Applications to Materials Science, Chemistry, and Biology*. (Birkhäuser Boston, 1999).
